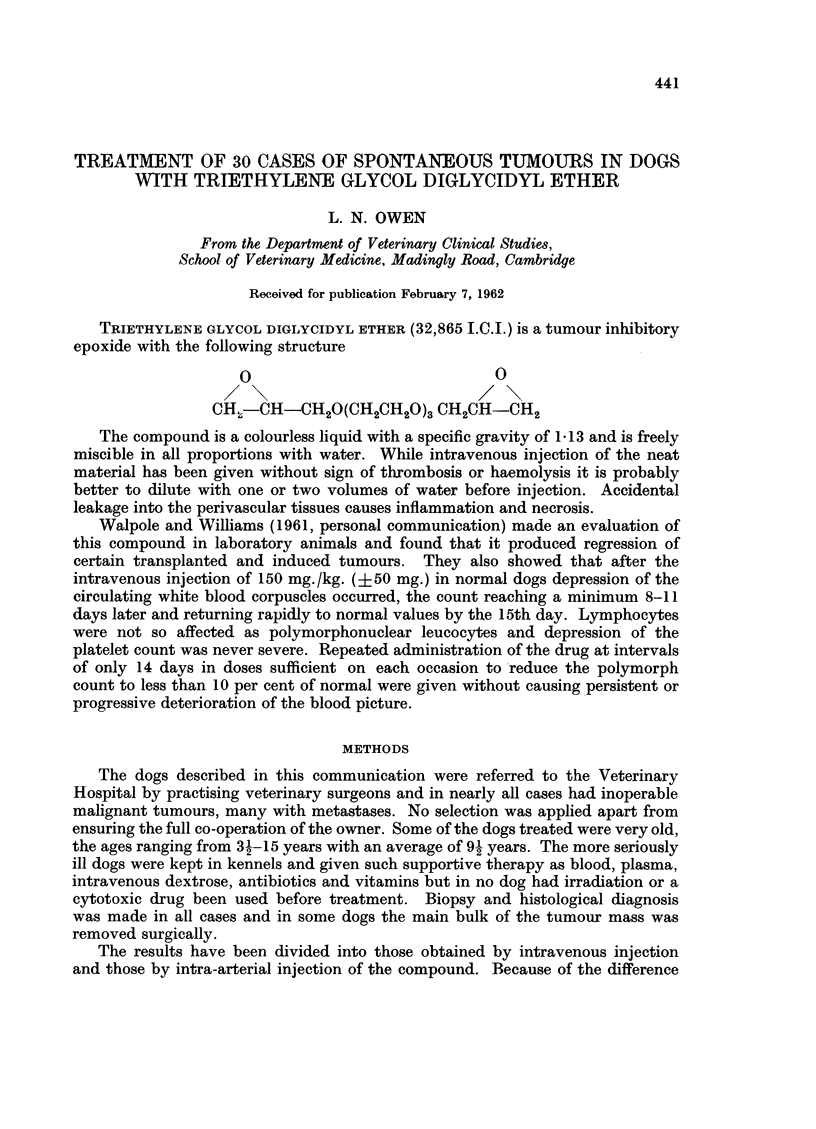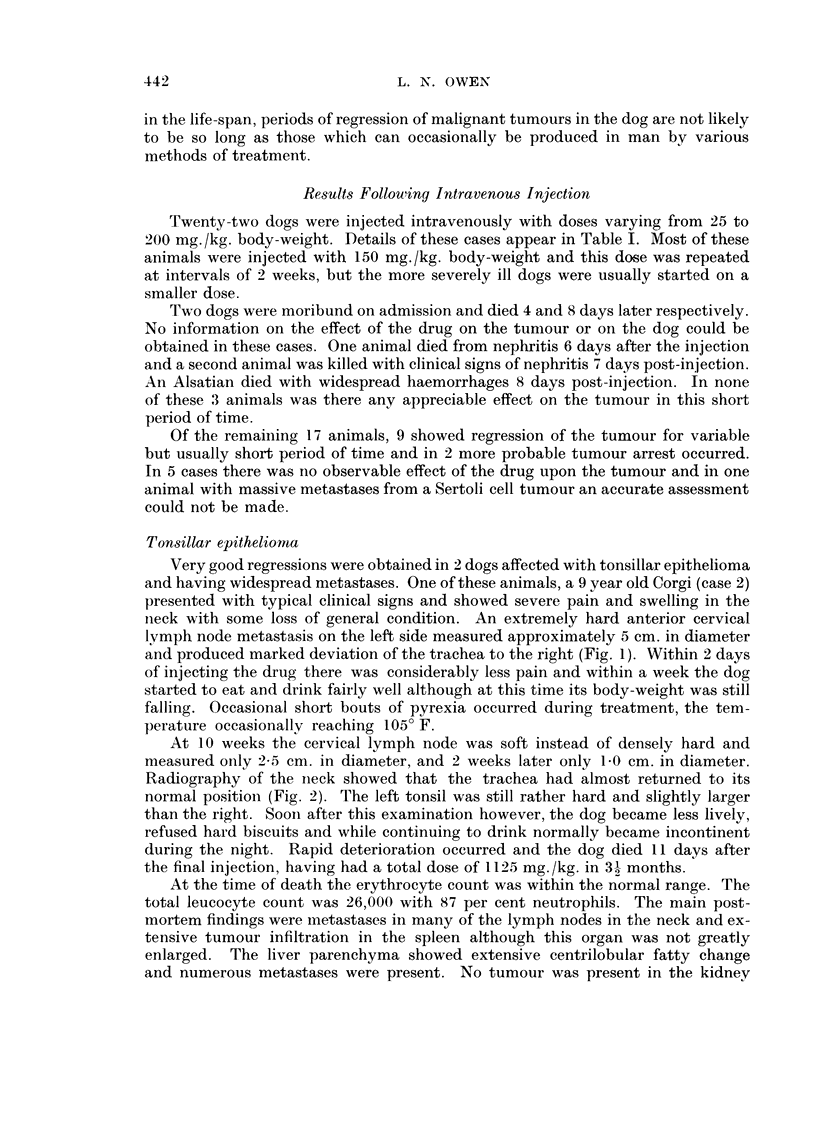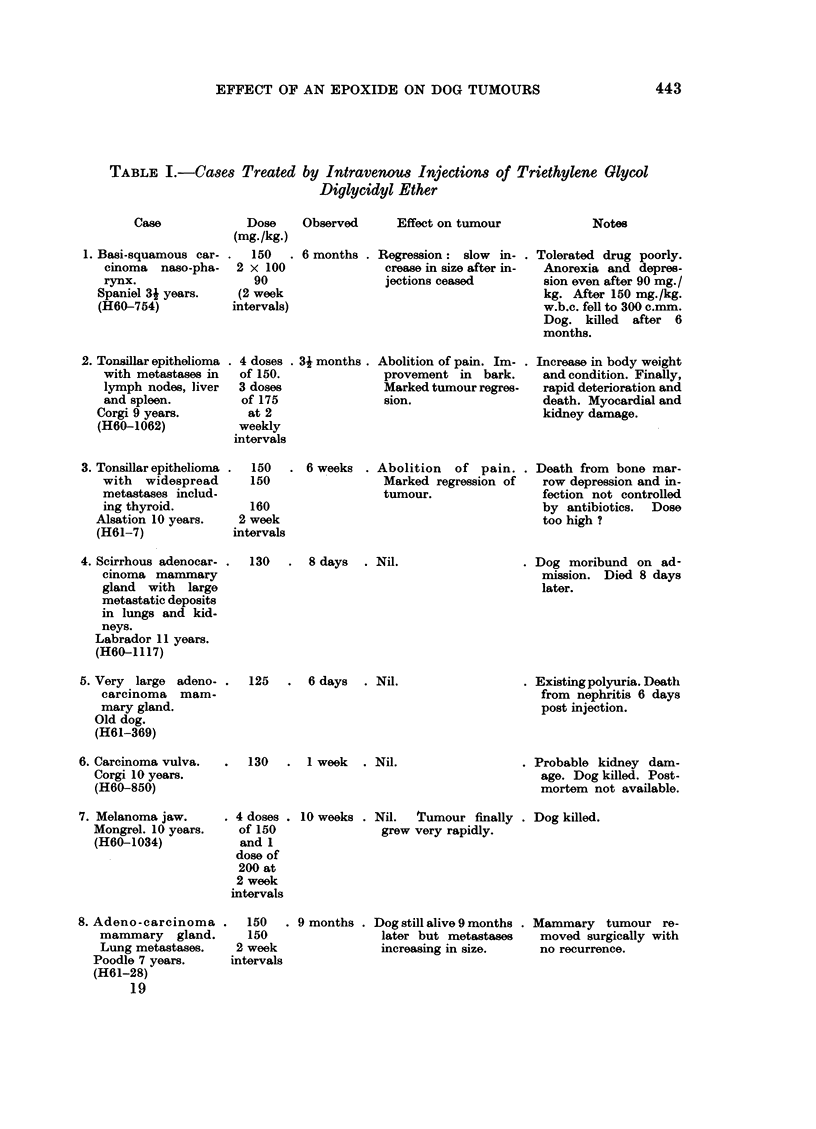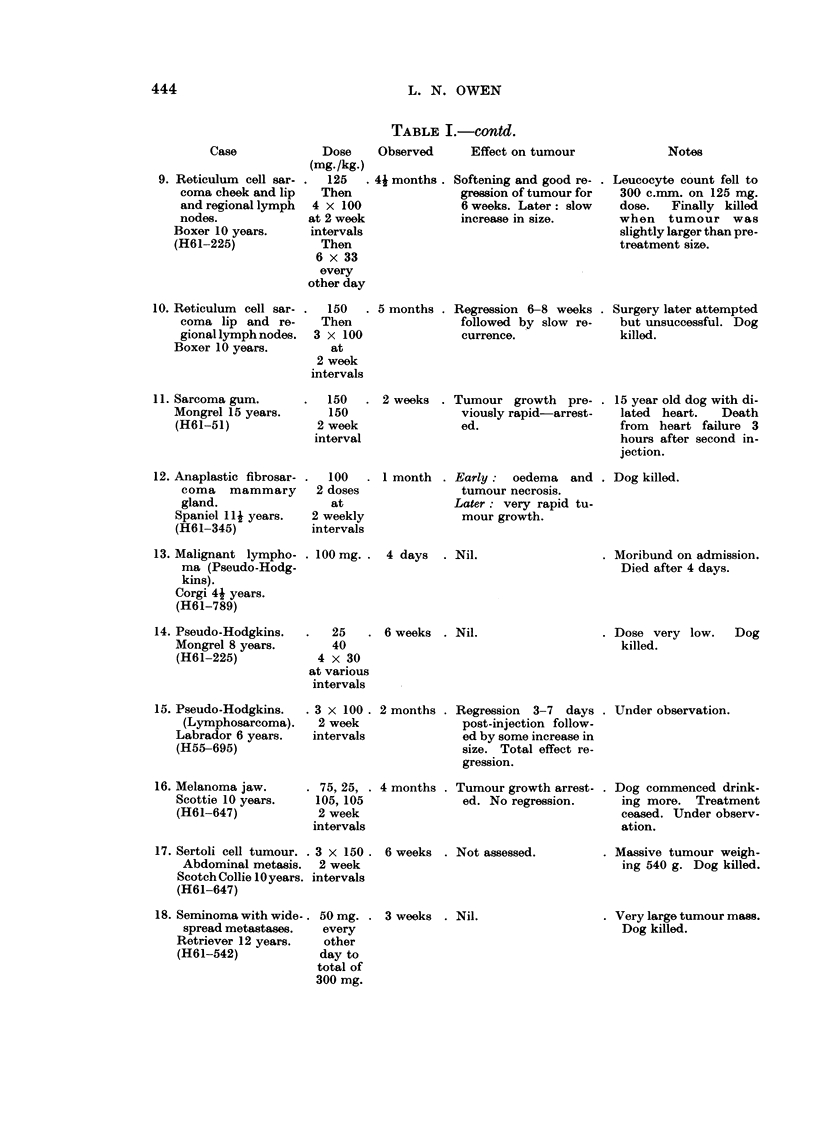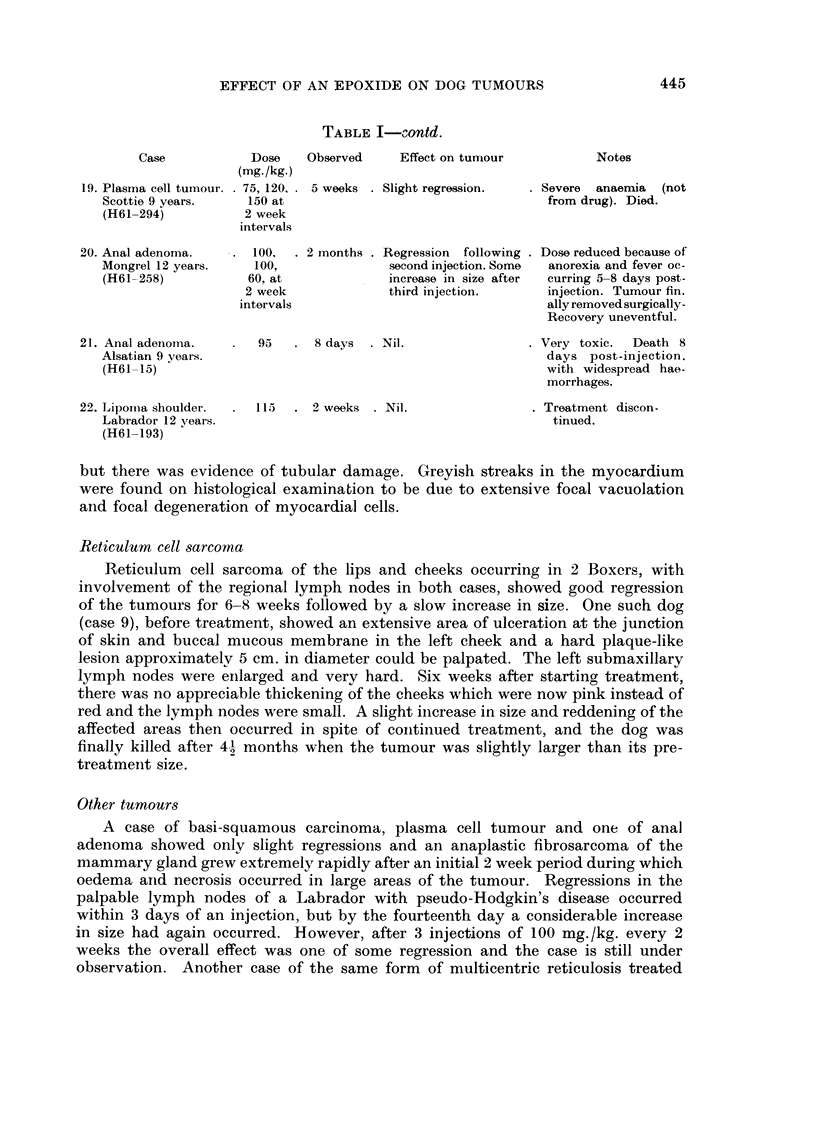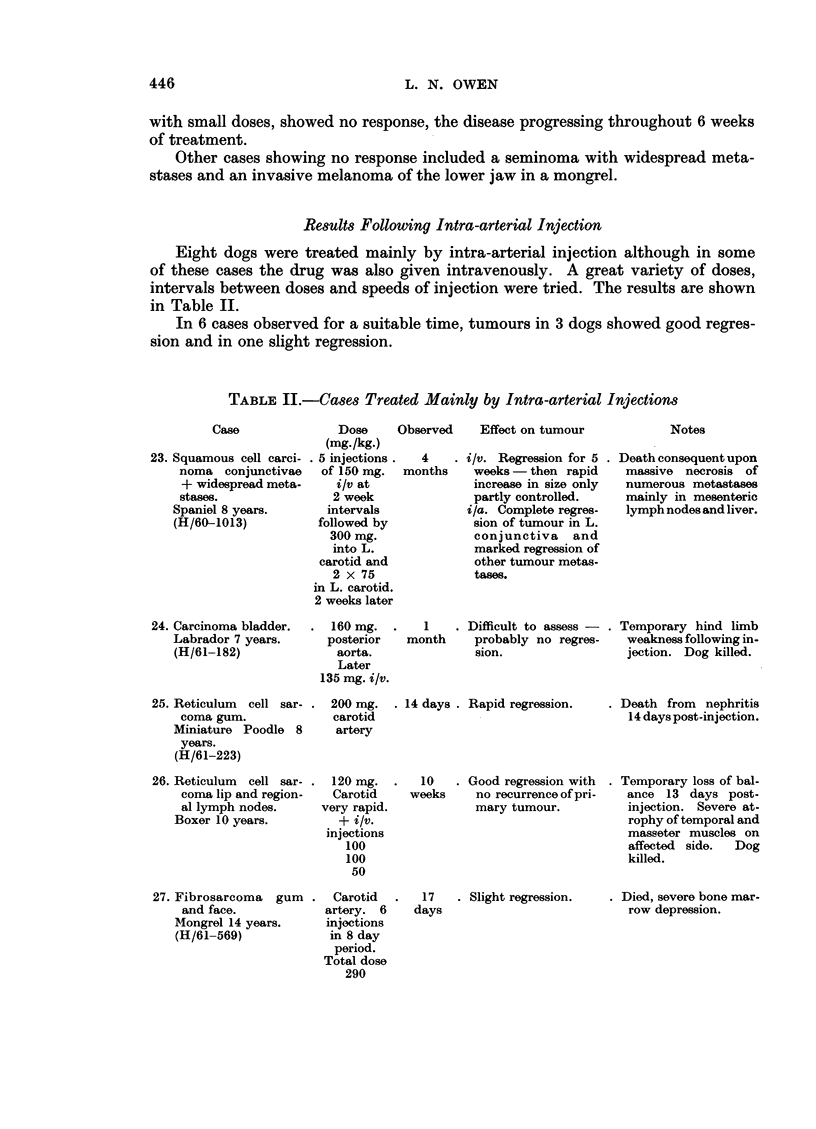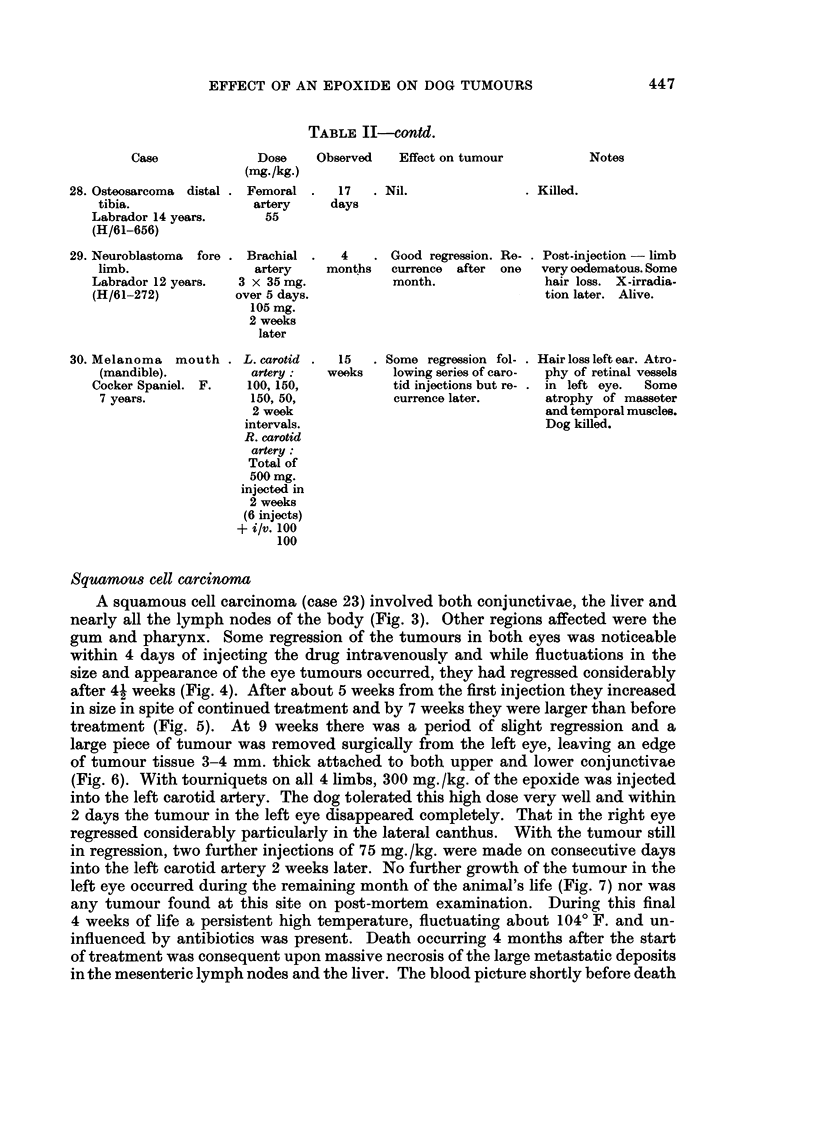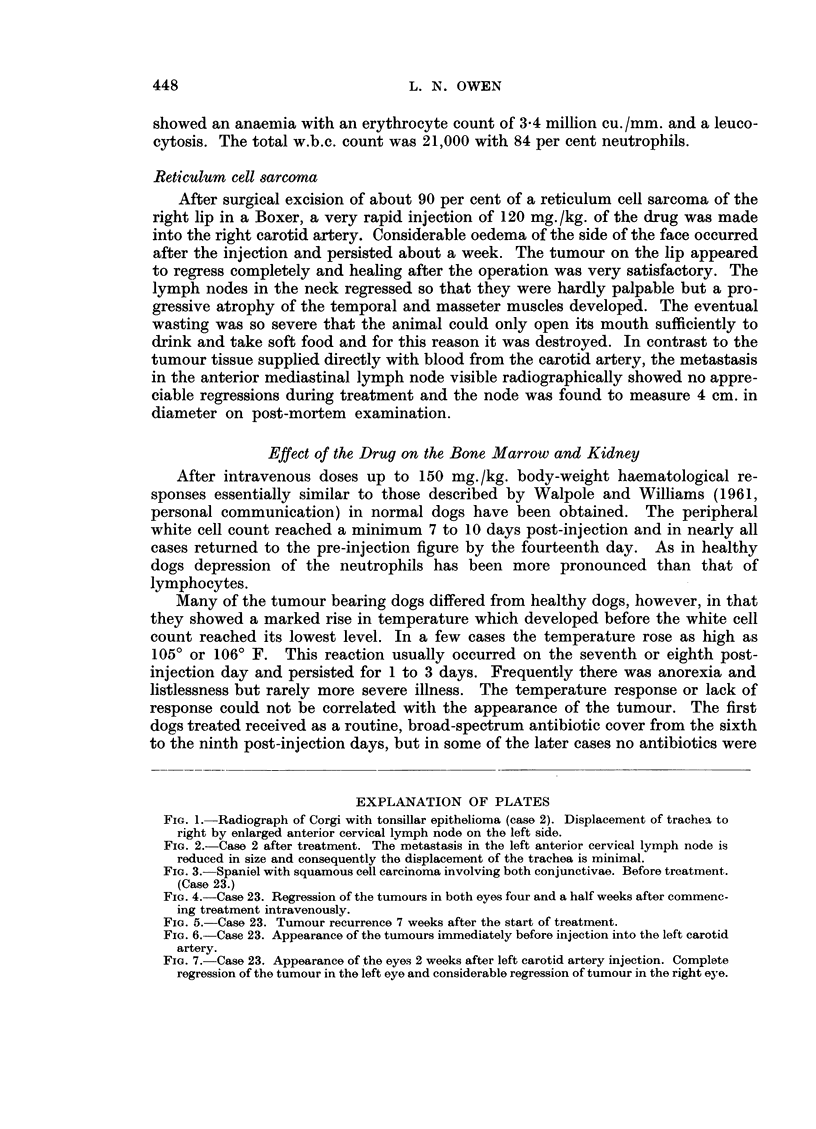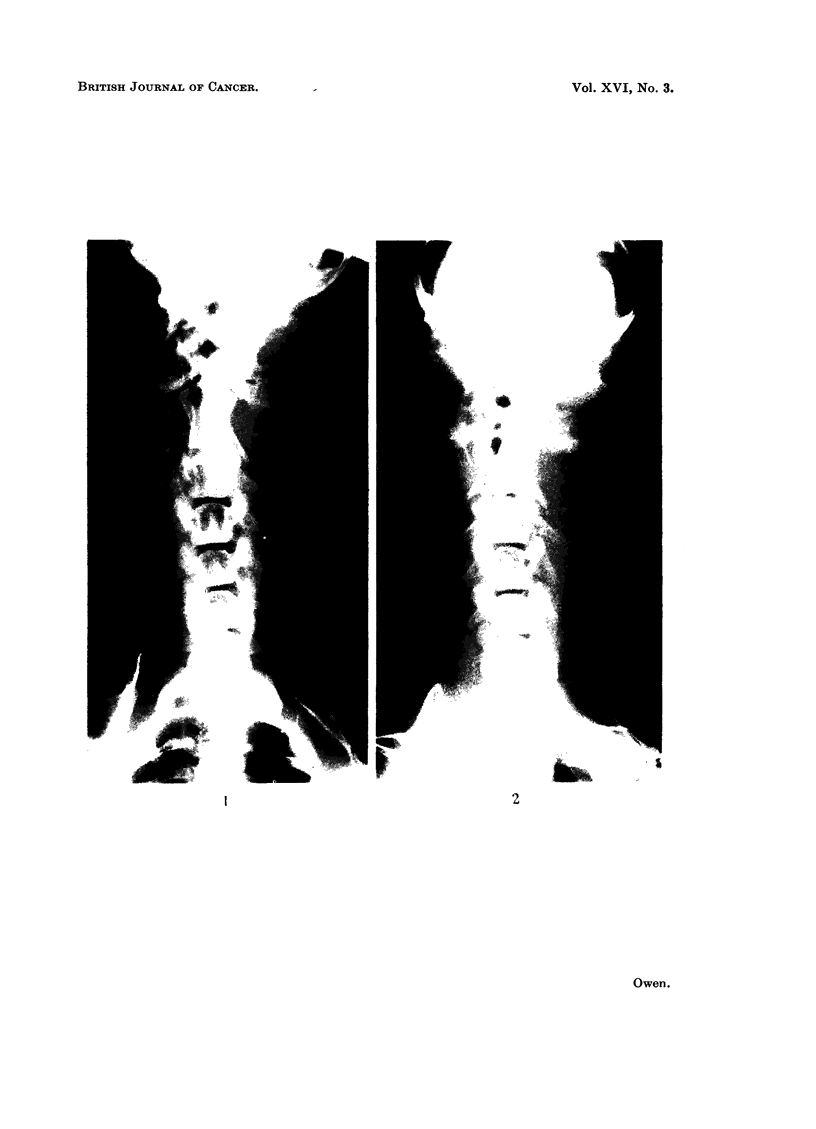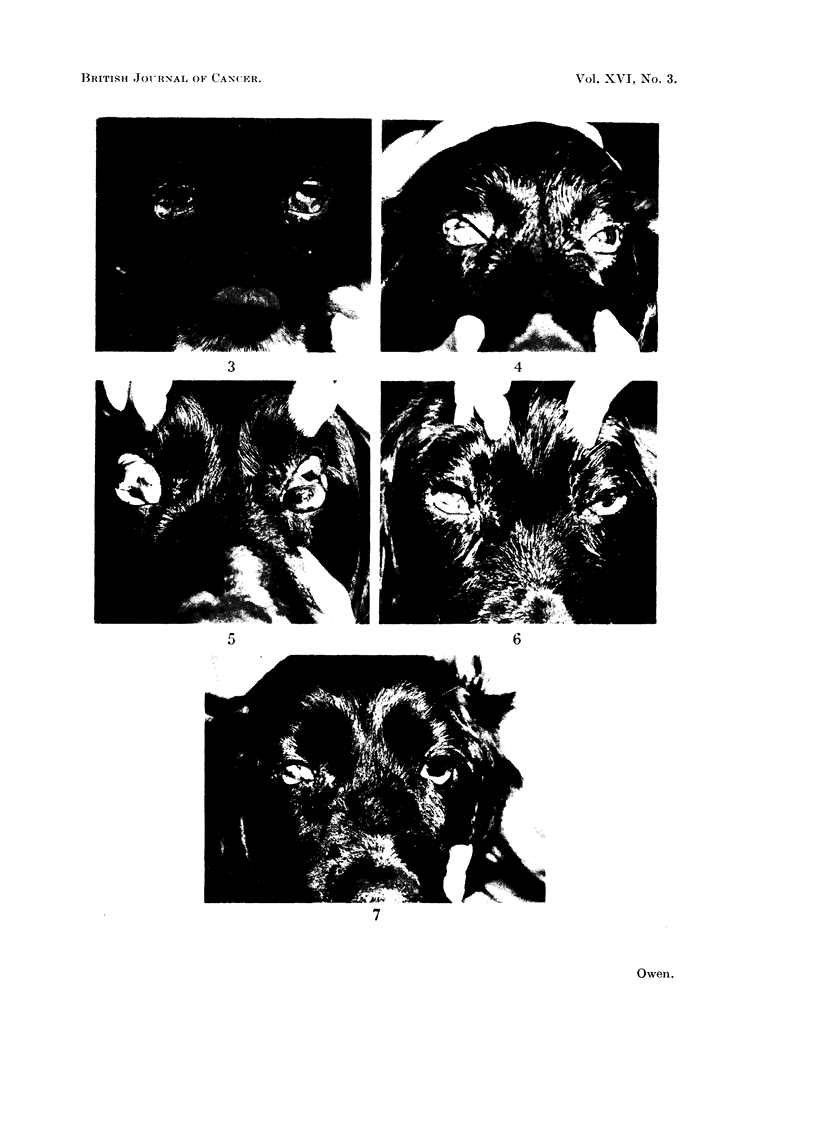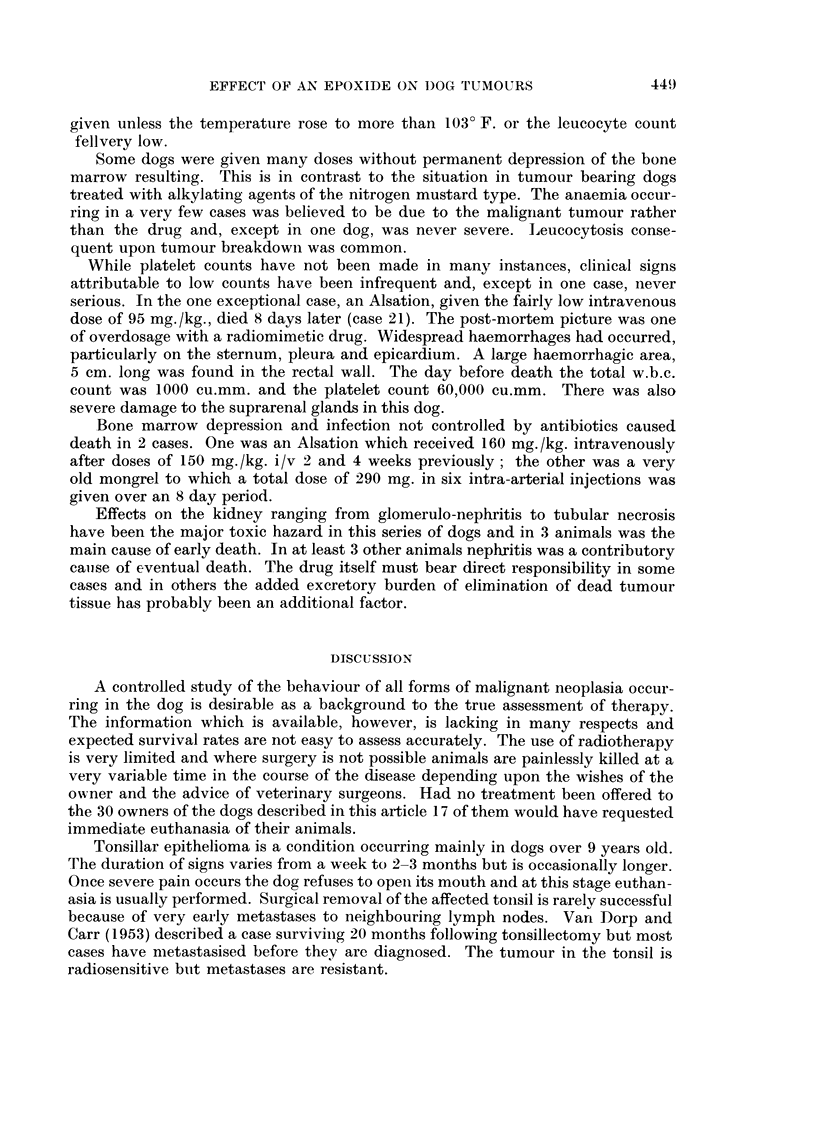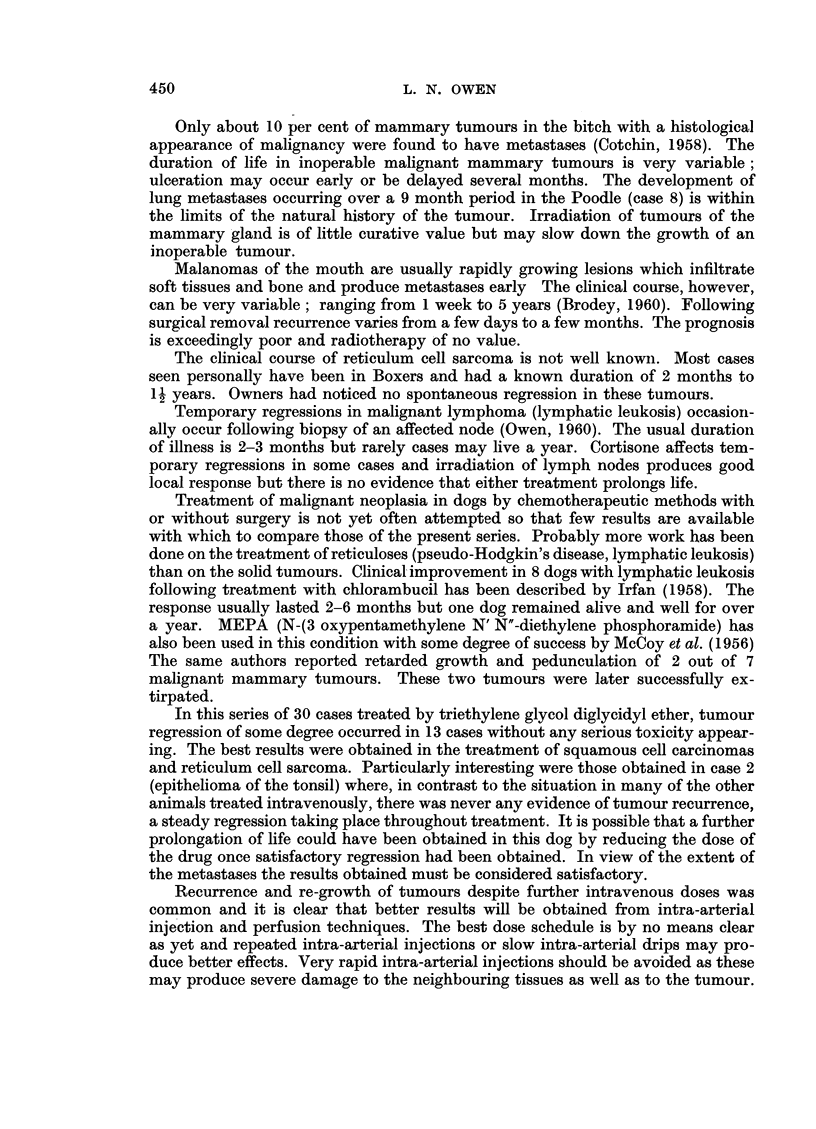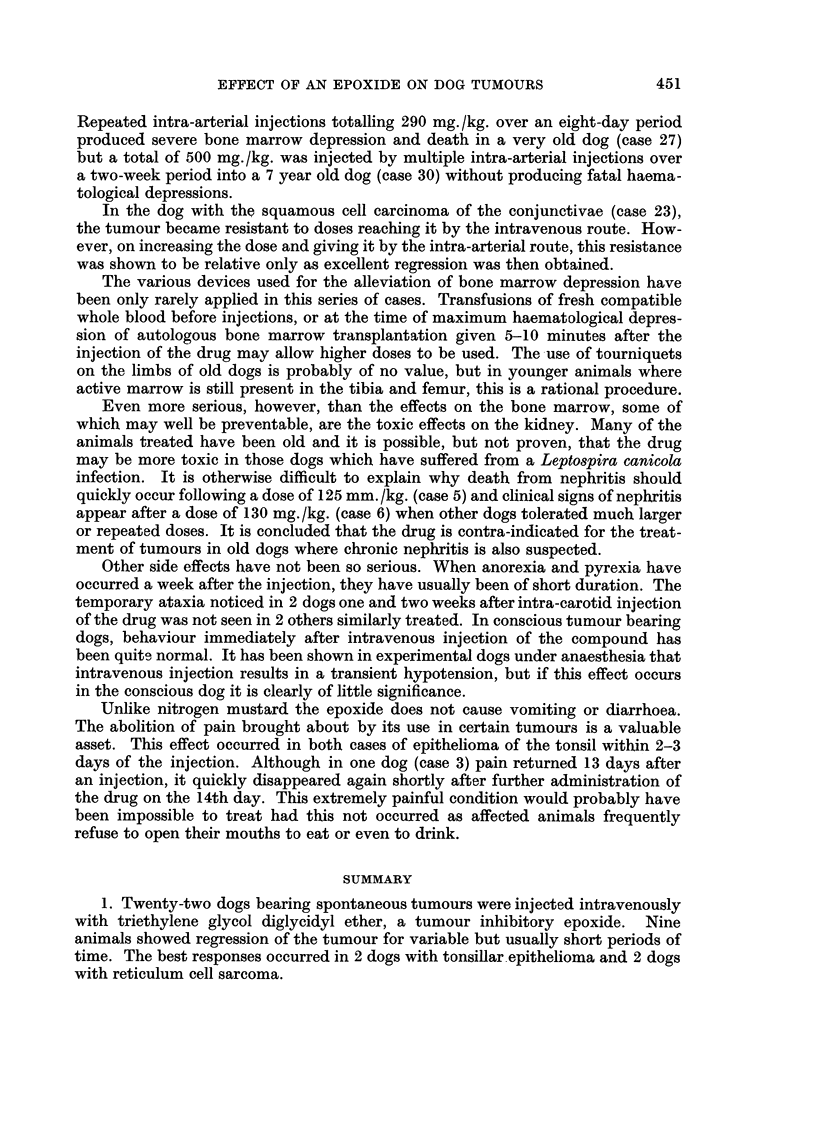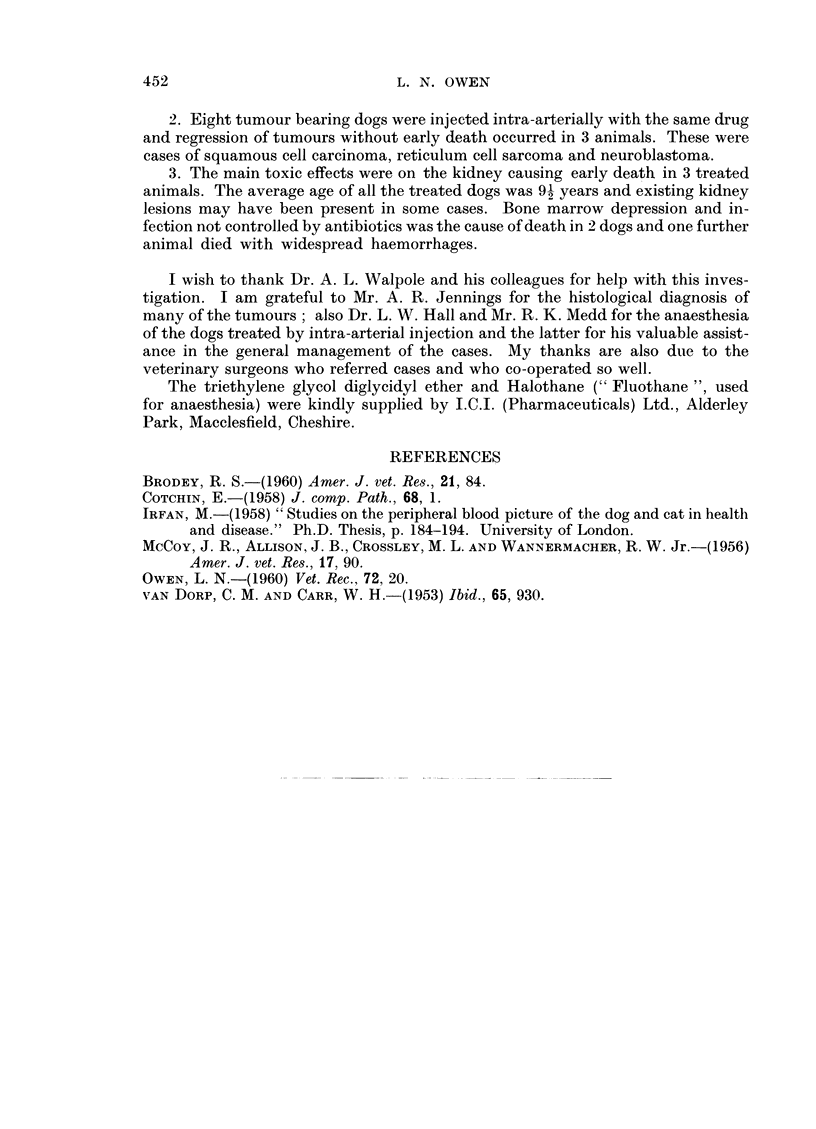# Treatment of 30 Cases of Spontaneous Tumours in Dogs with Triethylene Glycol Diglycidyl Ether

**DOI:** 10.1038/bjc.1962.49

**Published:** 1962-09

**Authors:** L. N. Owen

## Abstract

**Images:**


### TREATMENT OF 30 CASES OF SPONTANEOUS TUMOURS IN DOGS WITH TRIETHYLENE GLYCOL DIGLYCIDYL ETHER

L. N. OWEN

*From the Department of Veterinary Clinical Studies, School of Veterinary Medicine, Madingly Road, Cambridge*

Received for publication February 7, 1962

TRIETHYLENE GLYCOL DIGLYCIDYL ETHER (32,865 I.C.I.) is a tumour inhibitory epoxide with the following structure

The compound is a colourless liquid with a specific gravity of 1·13 and is freely miscible in all proportions with water. While intravenous injection of the neat material has been given without sign of thrombosis or haemolysis it is probably better to dilute with one or two volumes of water before injection. Accidental leakage into the perivascular tissues causes inflammation and necrosis.

Walpole and Williams (1961, personal communication) made an evaluation of this compound in laboratory animals and found that it produced regression of certain transplanted and induced tumours. They also showed that after the intravenous injection of 150 mg./kg. ($\pm$50 mg.) in normal dogs depression of the circulating white blood corpuscles occurred, the count reaching a minimum 8–11 days later and returning rapidly to normal values by the 15th day. Lymphocytes were not so affected as polymorphonuclear leucocytes and depression of the platelet count was never severe. Repeated administration of the drug at intervals of only 14 days in doses sufficient on each occasion to reduce the polymorph count to less than 10 per cent of normal were given without causing persistent or progressive deterioration of the blood picture.

### METHODS

The dogs described in this communication were referred to the Veterinary Hospital by practising veterinary surgeons and in nearly all cases had inoperable malignant tumours, many with metastases. No selection was applied apart from ensuring the full co-operation of the owner. Some of the dogs treated were very old, the ages ranging from 3½–15 years with an average of 9½ years. The more seriously ill dogs were kept in kennels and given such supportive therapy as blood, plasma, intravenous dextrose, antibiotics and vitamins but in no dog had irradiation or a cytotoxic drug been used before treatment. Biopsy and histological diagnosis was made in all cases and in some dogs the main bulk of the tumour mass was removed surgically.

The results have been divided into those obtained by intravenous injection and those by intra-arterial injection of the compound. Because of the difference in the life-span, periods of regression of malignant tumours in the dog are not likely to be so long as those which can occasionally be produced in man by various methods of treatment.

*Results Following Intravenous Injection*

Twenty-two dogs were injected intravenously with doses varying from 25 to 200 mg./kg. body-weight. Details of these cases appear in Table I. Most of these animals were injected with 150 mg./kg. body-weight and this dose was repeated at intervals of 2 weeks, but the more severely ill dogs were usually started on a smaller dose.

Two dogs were moribund on admission and died 4 and 8 days later respectively. No information on the effect of the drug on the tumour or on the dog could be obtained in these cases. One animal died from nephritis 6 days after the injection and a second animal was killed with clinical signs of nephritis 7 days post-injection. An Alsatian died with widespread haemorrhages 8 days post-injection. In none of these 3 animals was there any appreciable effect on the tumour in this short period of time.

Of the remaining 17 animals, 9 showed regression of the tumour for variable but usually short period of time and in 2 more probable tumour arrest occurred. In 5 cases there was no observable effect of the drug upon the tumour and in one animal with massive metastases from a Sertoli cell tumour an accurate assessment could not be made.

*Tonsillar epithelioma*

Very good regressions were obtained in 2 dogs affected with tonsillar epithelioma and having widespread metastases. One of these animals, a 9 year old Corgi (case 2) presented with typical clinical signs and showed severe pain and swelling in the neck with some loss of general condition. An extremely hard anterior cervical lymph node metastasis on the left side measured approximately 5 cm. in diameter and produced marked deviation of the trachea to the right (Fig. 1). Within 2 days of injecting the drug there was considerably less pain and within a week the dog started to eat and drink fairly well although at this time its body-weight was still falling. Occasional short bouts of pyrexia occurred during treatment, the temperature occasionally reaching 105° F.

At 10 weeks the cervical lymph node was soft instead of densely hard and measured only 2·5 cm. in diameter, and 2 weeks later only 1·0 cm. in diameter. Radiography of the neck showed that the trachea had almost returned to its normal position (Fig. 2). The left tonsil was still rather hard and slightly larger than the right. Soon after this examination however, the dog became less lively, refused hard biscuits and while continuing to drink normally became incontinent during the night. Rapid deterioration occurred and the dog died 11 days after the final injection, having had a total dose of 1125 mg./kg. in $3\frac{1}{2}$ months.

At the time of death the erythrocyte count was within the normal range. The total leucocyte count was 26,000 with 87 per cent neutrophils. The main post-mortem findings were metastases in many of the lymph nodes in the neck and extensive tumour infiltration in the spleen although this organ was not greatly enlarged. The liver parenchyma showed extensive centrilobular fatty change and numerous metastases were present. No tumour was present in the kidney

TABLE I.—*Cases Treated by Intravenous Injections of Triethylene Glycol Diglycidyl Ether*

| Case | Dose (mg./kg.) | Observed | Effect on tumour | Notes |
|---|---|---|---|---|
| 1. Basi-squamous carcinoma naso-pharynx. Spaniel 3½ years. (H60–754) | 150<br>2 × 100<br>90<br>(2 week intervals) | 6 months | Regression: slow increase in size after injections ceased | Tolerated drug poorly. Anorexia and depression even after 90 mg./kg. After 150 mg./kg. w.b.c. fell to 300 c.mm. Dog. killed after 6 months. |
| 2. Tonsillar epithelioma with metastases in lymph nodes, liver and spleen. Corgi 9 years. (H60–1062) | 4 doses of 150. 3 doses of 175 at 2 weekly intervals | 3½ months | Abolition of pain. Improvement in bark. Marked tumour regression. | Increase in body weight and condition. Finally, rapid deterioration and death. Myocardial and kidney damage. |
| 3. Tonsillar epithelioma with widespread metastases including thyroid. Alsation 10 years. (H61–7) | 150<br>150<br>160<br>2 week intervals | 6 weeks | Abolition of pain. Marked regression of tumour. | Death from bone marrow depression and infection not controlled by antibiotics. Dose too high ? |
| 4. Scirrhous adenocarcinoma mammary gland with large metastatic deposits in lungs and kidneys. Labrador 11 years. (H60–1117) | 130 | 8 days | Nil. | Dog moribund on admission. Died 8 days later. |
| 5. Very large adenocarcinoma mammary gland. Old dog. (H61–369) | 125 | 6 days | Nil. | Existing polyuria. Death from nephritis 6 days post injection. |
| 6. Carcinoma vulva. Corgi 10 years. (H60–850) | 130 | 1 week | Nil. | Probable kidney damage. Dog killed. Postmortem not available. |
| 7. Melanoma jaw. Mongrel. 10 years. (H60–1034) | 4 doses of 150 and 1 dose of 200 at 2 week intervals | 10 weeks | Nil. Tumour finally grew very rapidly. | Dog killed. |
| 8. Adeno-carcinoma mammary gland. Lung metastases. Poodle 7 years. (H61–28) | 150<br>150<br>2 week intervals | 9 months | Dog still alive 9 months later but metastases increasing in size. | Mammary tumour removed surgically with no recurrence. |

TABLE I.—*contd.*

| Case | Dose (mg./kg.) | Observed | Effect on tumour | Notes |
|---|---|---|---|---|
| 9. Reticulum cell sarcoma cheek and lip and regional lymph nodes. Boxer 10 years. (H61–225) | 125 Then 4 × 100 at 2 week intervals Then 6 × 33 every other day | 4½ months | Softening and good regression of tumour for 6 weeks. Later: slow increase in size. | Leucocyte count fell to 300 c.mm. on 125 mg. dose. Finally killed when tumour was slightly larger than pretreatment size. |
| 10. Reticulum cell sarcoma lip and regional lymph nodes. Boxer 10 years. | 150 Then 3 × 100 at 2 week intervals | 5 months | Regression 6–8 weeks followed by slow recurrence. | Surgery later attempted but unsuccessful. Dog killed. |
| 11. Sarcoma gum. Mongrel 15 years. (H61–51) | 150 150 2 week interval | 2 weeks | Tumour growth previously rapid—arrested. | 15 year old dog with dilated heart. Death from heart failure 3 hours after second injection. |
| 12. Anaplastic fibrosarcoma mammary gland. Spaniel 11½ years. (H61–345) | 100 2 doses at 2 weekly intervals | 1 month | *Early:* oedema and tumour necrosis. *Later:* very rapid tumour growth. | Dog killed. |
| 13. Malignant lymphoma (Pseudo-Hodgkins). Corgi 4½ years. (H61–789) | 100 mg. | 4 days | Nil. | Moribund on admission. Died after 4 days. |
| 14. Pseudo-Hodgkins. Mongrel 8 years. (H61–225) | 25 40 4 × 30 at various intervals | 6 weeks | Nil. | Dose very low. Dog killed. |
| 15. Pseudo-Hodgkins. (Lymphosarcoma). Labrador 6 years. (H55–695) | 3 × 100 2 week intervals | 2 months | Regression 3–7 days post-injection followed by some increase in size. Total effect regression. | Under observation. |
| 16. Melanoma jaw. Scottie 10 years. (H61–647) | 75, 25, 105, 105 2 week intervals | 4 months | Tumour growth arrested. No regression. | Dog commenced drinking more. Treatment ceased. Under observation. |
| 17. Sertoli cell tumour. Abdominal metasis. Scotch Collie 10 years. (H61–647) | 3 × 150 2 week intervals | 6 weeks | Not assessed. | Massive tumour weighing 540 g. Dog killed. |
| 18. Seminoma with widespread metastases. Retriever 12 years. (H61–542) | 50 mg. every other day to total of 300 mg. | 3 weeks | Nil. | Very large tumour mass. Dog killed. |

TABLE I—*contd.*

| Case | Dose (mg./kg.) | Observed | Effect on tumour | Notes |
|---|---|---|---|---|
| 19. Plasma cell tumour. Scottie 9 years. (H61–294) | 75, 120, 150 at 2 week intervals | 5 weeks | Slight regression. | Severe anaemia (not from drug). Died. |
| 20. Anal adenoma. Mongrel 12 years. (H61–258) | 100, 100, 60, at 2 week intervals | 2 months | Regression following second injection. Some increase in size after third injection. | Dose reduced because of anorexia and fever occurring 5–8 days post-injection. Tumour finally removed surgically. Recovery uneventful. |
| 21. Anal adenoma. Alsatian 9 years. (H61–15) | 95 | 8 days | Nil. | Very toxic. Death 8 days post-injection, with widespread haemorrhages. |
| 22. Lipoma shoulder. Labrador 12 years. (H61–193) | 115 | 2 weeks | Nil. | Treatment discontinued. |

but there was evidence of tubular damage. Greyish streaks in the myocardium were found on histological examination to be due to extensive focal vacuolation and focal degeneration of myocardial cells.

*Reticulum cell sarcoma*

Reticulum cell sarcoma of the lips and cheeks occurring in 2 Boxers, with involvement of the regional lymph nodes in both cases, showed good regression of the tumours for 6–8 weeks followed by a slow increase in size. One such dog (case 9), before treatment, showed an extensive area of ulceration at the junction of skin and buccal mucous membrane in the left cheek and a hard plaque-like lesion approximately 5 cm. in diameter could be palpated. The left submaxillary lymph nodes were enlarged and very hard. Six weeks after starting treatment, there was no appreciable thickening of the cheeks which were now pink instead of red and the lymph nodes were small. A slight increase in size and reddening of the affected areas then occurred in spite of continued treatment, and the dog was finally killed after 4½ months when the tumour was slightly larger than its pre-treatment size.

*Other tumours*

A case of basi-squamous carcinoma, plasma cell tumour and one of anal adenoma showed only slight regressions and an anaplastic fibrosarcoma of the mammary gland grew extremely rapidly after an initial 2 week period during which oedema and necrosis occurred in large areas of the tumour. Regressions in the palpable lymph nodes of a Labrador with pseudo-Hodgkin's disease occurred within 3 days of an injection, but by the fourteenth day a considerable increase in size had again occurred. However, after 3 injections of 100 mg./kg. every 2 weeks the overall effect was one of some regression and the case is still under observation. Another case of the same form of multicentric reticulosis treated with small doses, showed no response, the disease progressing throughout 6 weeks of treatment.

Other cases showing no response included a seminoma with widespread metastases and an invasive melanoma of the lower jaw in a mongrel.

### *Results Following Intra-arterial Injection*

Eight dogs were treated mainly by intra-arterial injection although in some of these cases the drug was also given intravenously. A great variety of doses, intervals between doses and speeds of injection were tried. The results are shown in Table II.

In 6 cases observed for a suitable time, tumours in 3 dogs showed good regression and in one slight regression.

TABLE II.—*Cases Treated Mainly by Intra-arterial Injections*

| Case | Dose (mg./kg.) | Observed | Effect on tumour | Notes |
|---|---|---|---|---|
| 23. Squamous cell carcinoma conjunctivae + widespread metastases. Spaniel 8 years. (H/60–1013) | 5 injections of 150 mg. *i/v* at 2 week intervals followed by 300 mg. into L. carotid and 2 × 75 in L. carotid. 2 weeks later | 4 months | *i/v.* Regression for 5 weeks — then rapid increase in size only partly controlled. *i/a.* Complete regression of tumour in L. conjunctiva and marked regression of other tumour metastases. | Death consequent upon massive necrosis of numerous metastases mainly in mesenteric lymph nodes and liver. |
| 24. Carcinoma bladder. Labrador 7 years. (H/61–182) | 160 mg. posterior aorta. Later 135 mg. *i/v.* | 1 month | Difficult to assess — probably no regression. | Temporary hind limb weakness following injection. Dog killed. |
| 25. Reticulum cell sarcoma gum. Miniature Poodle 8 years. (H/61–223) | 200 mg. carotid artery | 14 days | Rapid regression. | Death from nephritis 14 days post-injection. |
| 26. Reticulum cell sarcoma lip and regional lymph nodes. Boxer 10 years. | 120 mg. Carotid very rapid. + *i/v.* injections 100 100 50 | 10 weeks | Good regression with no recurrence of primary tumour. | Temporary loss of balance 13 days post-injection. Severe atrophy of temporal and masseter muscles on affected side. Dog killed. |
| 27. Fibrosarcoma gum and face. Mongrel 14 years. (H/61–569) | Carotid artery. 6 injections in 8 day period. Total dose 290 | 17 days | Slight regression. | Died, severe bone marrow depression. |

TABLE II—*contd.*

| Case | Dose (mg./kg.) | Observed | Effect on tumour | Notes |
|---|---|---|---|---|
| 28. Osteosarcoma distal tibia. Labrador 14 years. (H/61–656) | Femoral artery 55 | 17 days | Nil. | Killed. |
| 29. Neuroblastoma fore limb. Labrador 12 years. (H/61–272) | Brachial artery 3 × 35 mg. over 5 days. 105 mg. 2 weeks later | 4 months | Good regression. Recurrence after one month. | Post-injection — limb very oedematous. Some hair loss. X-irradiation later. Alive. |
| 30. Melanoma mouth (mandible). Cocker Spaniel. F. 7 years. | *L. carotid artery :* 100, 150, 150, 50, 2 week intervals. *R. carotid artery :* Total of 500 mg. injected in 2 weeks (6 injects) + *i/v.* 100 100 | 15 weeks | Some regression following series of carotid injections but recurrence later. | Hair loss left ear. Atrophy of retinal vessels in left eye. Some atrophy of masseter and temporal muscles. Dog killed. |

*Squamous cell carcinoma*

A squamous cell carcinoma (case 23) involved both conjunctivae, the liver and nearly all the lymph nodes of the body (Fig. 3). Other regions affected were the gum and pharynx. Some regression of the tumours in both eyes was noticeable within 4 days of injecting the drug intravenously and while fluctuations in the size and appearance of the eye tumours occurred, they had regressed considerably after 4½ weeks (Fig. 4). After about 5 weeks from the first injection they increased in size in spite of continued treatment and by 7 weeks they were larger than before treatment (Fig. 5). At 9 weeks there was a period of slight regression and a large piece of tumour was removed surgically from the left eye, leaving an edge of tumour tissue 3–4 mm. thick attached to both upper and lower conjunctivae (Fig. 6). With tourniquets on all 4 limbs, 300 mg./kg. of the epoxide was injected into the left carotid artery. The dog tolerated this high dose very well and within 2 days the tumour in the left eye disappeared completely. That in the right eye regressed considerably particularly in the lateral canthus. With the tumour still in regression, two further injections of 75 mg./kg. were made on consecutive days into the left carotid artery 2 weeks later. No further growth of the tumour in the left eye occurred during the remaining month of the animal's life (Fig. 7) nor was any tumour found at this site on post-mortem examination. During this final 4 weeks of life a persistent high temperature, fluctuating about 104° F. and uninfluenced by antibiotics was present. Death occurring 4 months after the start of treatment was consequent upon massive necrosis of the large metastatic deposits in the mesenteric lymph nodes and the liver. The blood picture shortly before death showed an anaemia with an erythrocyte count of 3·4 million cu./mm. and a leucocytosis. The total w.b.c. count was 21,000 with 84 per cent neutrophils.

*Reticulum cell sarcoma*

After surgical excision of about 90 per cent of a reticulum cell sarcoma of the right lip in a Boxer, a very rapid injection of 120 mg./kg. of the drug was made into the right carotid artery. Considerable oedema of the side of the face occurred after the injection and persisted about a week. The tumour on the lip appeared to regress completely and healing after the operation was very satisfactory. The lymph nodes in the neck regressed so that they were hardly palpable but a progressive atrophy of the temporal and masseter muscles developed. The eventual wasting was so severe that the animal could only open its mouth sufficiently to drink and take soft food and for this reason it was destroyed. In contrast to the tumour tissue supplied directly with blood from the carotid artery, the metastasis in the anterior mediastinal lymph node visible radiographically showed no appreciable regressions during treatment and the node was found to measure 4 cm. in diameter on post-mortem examination.

### *Effect of the Drug on the Bone Marrow and Kidney*

After intravenous doses up to 150 mg./kg. body-weight haematological responses essentially similar to those described by Walpole and Williams (1961, personal communication) in normal dogs have been obtained. The peripheral white cell count reached a minimum 7 to 10 days post-injection and in nearly all cases returned to the pre-injection figure by the fourteenth day. As in healthy dogs depression of the neutrophils has been more pronounced than that of lymphocytes.

Many of the tumour bearing dogs differed from healthy dogs, however, in that they showed a marked rise in temperature which developed before the white cell count reached its lowest level. In a few cases the temperature rose as high as 105° or 106° F. This reaction usually occurred on the seventh or eighth post-injection day and persisted for 1 to 3 days. Frequently there was anorexia and listlessness but rarely more severe illness. The temperature response or lack of response could not be correlated with the appearance of the tumour. The first dogs treated received as a routine, broad-spectrum antibiotic cover from the sixth to the ninth post-injection days, but in some of the later cases no antibiotics were

---

EXPLANATION OF PLATES

FIG. 1.—Radiograph of Corgi with tonsillar epithelioma (case 2). Displacement of trachea to right by enlarged anterior cervical lymph node on the left side.

FIG. 2.—Case 2 after treatment. The metastasis in the left anterior cervical lymph node is reduced in size and consequently the displacement of the trachea is minimal.

FIG. 3.—Spaniel with squamous cell carcinoma involving both conjunctivae. Before treatment. (Case 23.)

FIG. 4.—Case 23. Regression of the tumours in both eyes four and a half weeks after commencing treatment intravenously.

FIG. 5.—Case 23. Tumour recurrence 7 weeks after the start of treatment.

FIG. 6.—Case 23. Appearance of the tumours immediately before injection into the left carotid artery.

FIG. 7.—Case 23. Appearance of the eyes 2 weeks after left carotid artery injection. Complete regression of the tumour in the left eye and considerable regression of tumour in the right eye.

1 2

Owen.

3

4

5

6

7

Owen.

given unless the temperature rose to more than 103° F. or the leucocyte count fellvery low.

Some dogs were given many doses without permanent depression of the bone marrow resulting. This is in contrast to the situation in tumour bearing dogs treated with alkylating agents of the nitrogen mustard type. The anaemia occurring in a very few cases was believed to be due to the malignant tumour rather than the drug and, except in one dog, was never severe. Leucocytosis consequent upon tumour breakdown was common.

While platelet counts have not been made in many instances, clinical signs attributable to low counts have been infrequent and, except in one case, never serious. In the one exceptional case, an Alsation, given the fairly low intravenous dose of 95 mg./kg., died 8 days later (case 21). The post-mortem picture was one of overdosage with a radiomimetic drug. Widespread haemorrhages had occurred, particularly on the sternum, pleura and epicardium. A large haemorrhagic area, 5 cm. long was found in the rectal wall. The day before death the total w.b.c. count was 1000 cu.mm. and the platelet count 60,000 cu.mm. There was also severe damage to the suprarenal glands in this dog.

Bone marrow depression and infection not controlled by antibiotics caused death in 2 cases. One was an Alsation which received 160 mg./kg. intravenously after doses of 150 mg./kg. i/v 2 and 4 weeks previously ; the other was a very old mongrel to which a total dose of 290 mg. in six intra-arterial injections was given over an 8 day period.

Effects on the kidney ranging from glomerulo-nephritis to tubular necrosis have been the major toxic hazard in this series of dogs and in 3 animals was the main cause of early death. In at least 3 other animals nephritis was a contributory cause of eventual death. The drug itself must bear direct responsibility in some cases and in others the added excretory burden of elimination of dead tumour tissue has probably been an additional factor.

### DISCUSSION

A controlled study of the behaviour of all forms of malignant neoplasia occurring in the dog is desirable as a background to the true assessment of therapy. The information which is available, however, is lacking in many respects and expected survival rates are not easy to assess accurately. The use of radiotherapy is very limited and where surgery is not possible animals are painlessly killed at a very variable time in the course of the disease depending upon the wishes of the owner and the advice of veterinary surgeons. Had no treatment been offered to the 30 owners of the dogs described in this article 17 of them would have requested immediate euthanasia of their animals.

Tonsillar epithelioma is a condition occurring mainly in dogs over 9 years old. The duration of signs varies from a week to 2–3 months but is occasionally longer. Once severe pain occurs the dog refuses to open its mouth and at this stage euthanasia is usually performed. Surgical removal of the affected tonsil is rarely successful because of very early metastases to neighbouring lymph nodes. Van Dorp and Carr (1953) described a case surviving 20 months following tonsillectomy but most cases have metastasised before they are diagnosed. The tumour in the tonsil is radiosensitive but metastases are resistant.

Only about 10 per cent of mammary tumours in the bitch with a histological appearance of malignancy were found to have metastases (Cotchin, 1958). The duration of life in inoperable malignant mammary tumours is very variable ; ulceration may occur early or be delayed several months. The development of lung metastases occurring over a 9 month period in the Poodle (case 8) is within the limits of the natural history of the tumour. Irradiation of tumours of the mammary gland is of little curative value but may slow down the growth of an inoperable tumour.

Malanomas of the mouth are usually rapidly growing lesions which infiltrate soft tissues and bone and produce metastases early The clinical course, however, can be very variable ; ranging from 1 week to 5 years (Brodey, 1960). Following surgical removal recurrence varies from a few days to a few months. The prognosis is exceedingly poor and radiotherapy of no value.

The clinical course of reticulum cell sarcoma is not well known. Most cases seen personally have been in Boxers and had a known duration of 2 months to $1\frac{1}{2}$ years. Owners had noticed no spontaneous regression in these tumours.

Temporary regressions in malignant lymphoma (lymphatic leukosis) occasionally occur following biopsy of an affected node (Owen, 1960). The usual duration of illness is 2–3 months but rarely cases may live a year. Cortisone affects temporary regressions in some cases and irradiation of lymph nodes produces good local response but there is no evidence that either treatment prolongs life.

Treatment of malignant neoplasia in dogs by chemotherapeutic methods with or without surgery is not yet often attempted so that few results are available with which to compare those of the present series. Probably more work has been done on the treatment of reticuloses (pseudo-Hodgkin's disease, lymphatic leukosis) than on the solid tumours. Clinical improvement in 8 dogs with lymphatic leukosis following treatment with chlorambucil has been described by Irfan (1958). The response usually lasted 2–6 months but one dog remained alive and well for over a year. MEPA (N-(3 oxypentamethylene N′ N″-diethylene phosphoramide) has also been used in this condition with some degree of success by McCoy *et al.* (1956) The same authors reported retarded growth and pedunculation of 2 out of 7 malignant mammary tumours. These two tumours were later successfully extirpated.

In this series of 30 cases treated by triethylene glycol diglycidyl ether, tumour regression of some degree occurred in 13 cases without any serious toxicity appearing. The best results were obtained in the treatment of squamous cell carcinomas and reticulum cell sarcoma. Particularly interesting were those obtained in case 2 (epithelioma of the tonsil) where, in contrast to the situation in many of the other animals treated intravenously, there was never any evidence of tumour recurrence, a steady regression taking place throughout treatment. It is possible that a further prolongation of life could have been obtained in this dog by reducing the dose of the drug once satisfactory regression had been obtained. In view of the extent of the metastases the results obtained must be considered satisfactory.

Recurrence and re-growth of tumours despite further intravenous doses was common and it is clear that better results will be obtained from intra-arterial injection and perfusion techniques. The best dose schedule is by no means clear as yet and repeated intra-arterial injections or slow intra-arterial drips may produce better effects. Very rapid intra-arterial injections should be avoided as these may produce severe damage to the neighbouring tissues as well as to the tumour.

Repeated intra-arterial injections totalling 290 mg./kg. over an eight-day period produced severe bone marrow depression and death in a very old dog (case 27) but a total of 500 mg./kg. was injected by multiple intra-arterial injections over a two-week period into a 7 year old dog (case 30) without producing fatal haematological depressions.

In the dog with the squamous cell carcinoma of the conjunctivae (case 23), the tumour became resistant to doses reaching it by the intravenous route. However, on increasing the dose and giving it by the intra-arterial route, this resistance was shown to be relative only as excellent regression was then obtained.

The various devices used for the alleviation of bone marrow depression have been only rarely applied in this series of cases. Transfusions of fresh compatible whole blood before injections, or at the time of maximum haematological depression of autologous bone marrow transplantation given 5–10 minutes after the injection of the drug may allow higher doses to be used. The use of tourniquets on the limbs of old dogs is probably of no value, but in younger animals where active marrow is still present in the tibia and femur, this is a rational procedure.

Even more serious, however, than the effects on the bone marrow, some of which may well be preventable, are the toxic effects on the kidney. Many of the animals treated have been old and it is possible, but not proven, that the drug may be more toxic in those dogs which have suffered from a *Leptospira canicola* infection. It is otherwise difficult to explain why death from nephritis should quickly occur following a dose of 125 mm./kg. (case 5) and clinical signs of nephritis appear after a dose of 130 mg./kg. (case 6) when other dogs tolerated much larger or repeated doses. It is concluded that the drug is contra-indicated for the treatment of tumours in old dogs where chronic nephritis is also suspected.

Other side effects have not been so serious. When anorexia and pyrexia have occurred a week after the injection, they have usually been of short duration. The temporary ataxia noticed in 2 dogs one and two weeks after intra-carotid injection of the drug was not seen in 2 others similarly treated. In conscious tumour bearing dogs, behaviour immediately after intravenous injection of the compound has been quite normal. It has been shown in experimental dogs under anaesthesia that intravenous injection results in a transient hypotension, but if this effect occurs in the conscious dog it is clearly of little significance.

Unlike nitrogen mustard the epoxide does not cause vomiting or diarrhoea. The abolition of pain brought about by its use in certain tumours is a valuable asset. This effect occurred in both cases of epithelioma of the tonsil within 2–3 days of the injection. Although in one dog (case 3) pain returned 13 days after an injection, it quickly disappeared again shortly after further administration of the drug on the 14th day. This extremely painful condition would probably have been impossible to treat had this not occurred as affected animals frequently refuse to open their mouths to eat or even to drink.

### SUMMARY

1. Twenty-two dogs bearing spontaneous tumours were injected intravenously with triethylene glycol diglycidyl ether, a tumour inhibitory epoxide. Nine animals showed regression of the tumour for variable but usually short periods of time. The best responses occurred in 2 dogs with tonsillar epithelioma and 2 dogs with reticulum cell sarcoma.

2. Eight tumour bearing dogs were injected intra-arterially with the same drug and regression of tumours without early death occurred in 3 animals. These were cases of squamous cell carcinoma, reticulum cell sarcoma and neuroblastoma.

3. The main toxic effects were on the kidney causing early death in 3 treated animals. The average age of all the treated dogs was $9\frac{1}{2}$ years and existing kidney lesions may have been present in some cases. Bone marrow depression and infection not controlled by antibiotics was the cause of death in 2 dogs and one further animal died with widespread haemorrhages.

I wish to thank Dr. A. L. Walpole and his colleagues for help with this investigation. I am grateful to Mr. A. R. Jennings for the histological diagnosis of many of the tumours ; also Dr. L. W. Hall and Mr. R. K. Medd for the anaesthesia of the dogs treated by intra-arterial injection and the latter for his valuable assistance in the general management of the cases. My thanks are also due to the veterinary surgeons who referred cases and who co-operated so well.

The triethylene glycol diglycidyl ether and Halothane (" Fluothane ", used for anaesthesia) were kindly supplied by I.C.I. (Pharmaceuticals) Ltd., Alderley Park, Macclesfield, Cheshire.

### REFERENCES

Brodey, R. S.—(1960) *Amer. J. vet. Res.*, **21**, 84.
Cotchin, E.—(1958) *J. comp. Path.*, **68**, 1.
Irfan, M.—(1958) " Studies on the peripheral blood picture of the dog and cat in health and disease." Ph.D. Thesis, p. 184–194. University of London.
McCoy, J. R., Allison, J. B., Crossley, M. L. and Wannermacher, R. W. Jr.—(1956) *Amer. J. vet. Res.*, **17**, 90.
Owen, L. N.—(1960) *Vet. Rec.*, **72**, 20.
van Dorp, C. M. and Carr, W. H.—(1953) *Ibid.*, **65**, 930.